# Health Risk Assessment of Metals in Antidiabetic Herbal Preparations: A Safety Screening

**DOI:** 10.1155/2024/6507185

**Published:** 2024-01-12

**Authors:** Nazmul Islam, Rausan Zamir, Md. Omar Faruque

**Affiliations:** ^1^Department of Nutrition and Food Technology, Jashore University of Science and Technology, Jashore, Bangladesh; ^2^Department of General Educational Development, Daffodil International University, Dhaka, Bangladesh; ^3^Department of Chemistry, University of Rajshahi, Rajshahi 6205, Bangladesh

## Abstract

The present study evaluates the human health risk of metals in locally consumed herbal preparations used to treat diabetes. Atomic absorption spectroscopy (AAS) was used after microwave-assisted digestion to mineralize the samples. Toxic metal assessment was done by adopting mathematical modeling for carcinogenic and noncarcinogenic risks in the exposed population and comparing the raw results with maximum residue limits (MRLs) set by regulatory authorities. Hazard quotient (HQ) values for Fe, Hg, Cu, Pb, and Zn were recorded above 1. Noncarcinogenic health risks remain in 29% of samples for Fe, 67% of samples for Hg, 17% of samples for Cu, 33% of samples for Pb, and 4% of samples for Zn. Hazard index (HI) values in 33% of samples were above 1. Carcinogenic risks for Pb, Cr, Cd, and Ni were higher than the acceptable limit (1 × 10^−6^). Carcinogenic health risks exist in 54% of samples for Pb, 58% of samples for Cr, 46% of samples for Cd, and 58% of samples for Ni. MRLs for metals were crossed in samples in varying degrees. This is a harrowing account and may put public health safety at risk. Considering these facts, there should be more investigation into toxic metals in other frequently marketed herbal drugs in the antidiabetic and other therapeutic classes. Pre- and postmarket monitoring strategies for the preparations should also be in place to ensure safe consumption.

## 1. Introduction

Diabetes is a chronic disease and is prevalent throughout the world [1]. Phytotherapy is the art of healing ailments by means of plant-based processes [2]. Remedy for ailments by means of plant-based therapeutics has been practiced since ancient times [3]. As an alternative remedy, consumption of antidiabetic herbal preparations is increasing [4].

However, increased consumption of the formulations does not safeguard drug safety [5], and doubt remains about their quality [6]. The scientific community presumed that the remedies may be reaching the consumer without going through proper inspections like their allopathic counterparts. Previous reports documented metal contamination in different herbal preparations [5–16].

Medicinal herbs pick up toxic metals from contaminated soils [17]. Toxic metal contamination is more likely when the herbal preparations are prepared in substandard industrial conditions and are transported in open-bed trucks, which exhaust pollutants from the ingredients [18]. There is also the possibility of the intentional addition of toxic metals to finished formulations, assuming their medicinal benefits. Metals have a tendency to accumulate in the food chain, and some of them could have damaging effects on human health even at very low concentrations due to their low excretion rates through the kidneys [19]. With increased intake above certain permissible limits, metals become toxic [10, 20]. Diseases such as cardiac dysfunction [21], gastrointestinal cancer [22], and impaired psychosocial and neurological behaviors [23, 24] set in due to chronic exposure to toxic metals at high concentrations.

Very few studies have been conducted on toxic metals in locally formulated herbal preparations, and most of the studies were under the current investigator [25, 26]. The city of Rajshahi forms an urban cluster with two nearby satellite towns, Nowhata and Katakhali. The city has a population of ten million. Rajshahi is the fourth most populous city in Bangladesh after Dhaka, Chattogram, and Khulna [27]. The consumption of antidiabetic herbal preparations for the management of different diseases is common among city dwellers. Although consumption requires proper inspection of these remedies from manufacturing to selling by the authority concerned, doubt remains with the formulations. As a result of the dubiety, drug safety may fall into pitfall.

### 1.1. Problem Statement

Research data on health risk in human health due to metal ingestion through antidiabetic herbal preparations by the local people are absent.

### 1.2. Research Gap

Risk evaluation considers the intake and consumption of the formulation. The health risk is mainly linked to the duration and rate of ingestion. At present, a major data gap in studies of herbal formulations is the considerable lack of intake (consumption and occurrence) data. For assessing the risk of pharmaceutical formulations, input assumptions such as concentration of metals in drugs, exposure duration, and frequency data are required. To the best of our knowledge, no investigation has been conducted on the carcinogenic and noncarcinogenic health risk assessments of the investigated samples.

### 1.3. Justification of the Study

Oral consumption of herbal drugs using “conservative” and “realistic” theoretical exposure scenarios results in mathematical risk modeling. The modeling uses HQ, HI, and ILCR values. Therefore, to obtain the health risk, the computation of HQ, HI, and ILCR should be performed [28]. This assessment enables to devise the intervention whether extra action is needed upon comparing risk results to cutoff points [29].

The findings as dissemination would help to create awareness in the scientific community and help policy makers take the right steps in ensuring herbal drug safety. This in turn would replenish the demand of United Nations (UN) Sustainable Development Goal (SDG) Goal No. 3 which vows to ensure “good health and wellbeing.”

## 2. Methodology

### 2.1. Study Design

**Figure 1:**
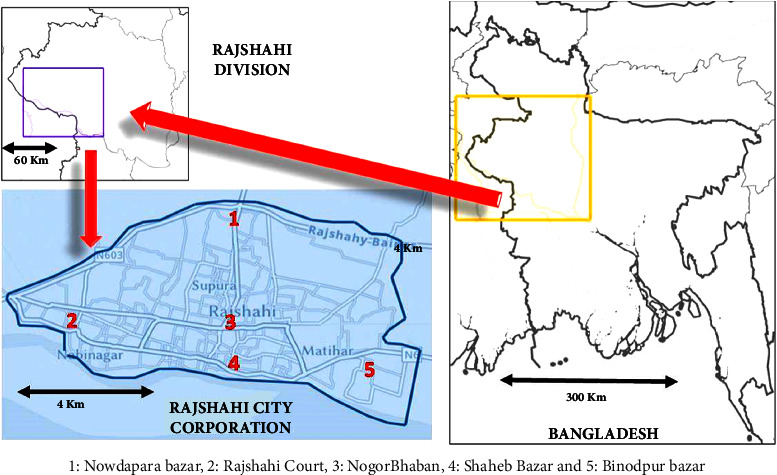
Sampling locations in Rajshahi City Corporation Area in Bangladesh.

The study was a cross-sectional observational study, where samples were collected from different sample collection hotspots (Figure 1). Samples were blindfolded by coding and preserved. Microwave digestion was performed for metal dissolution from the sample into diluents, followed by atomic absorption spectrometry (AAS). Metal concentration data were modeled and assessed for carcinogenic and noncarcinogenic health effects.

### 2.2. Study Area

The study area was restricted to the Rajshahi City Corporation area. Sampling was done at five sampling locations. Sampling locations were tagged with numerical values (Figure 1). All the investigated drugs were finished in commercial packs. The location of the drug-selling outlets bears the following resemblance:

1. Herbal drug-selling hotspots.
2. The areas were densely populated, and people bought drugs from the close vicinity of their residing location.
3. There is mass transferring of people through the areas selected, making the areas transferring a zone of people. This adds up to more people, and on their way to the city and upon returning home, they buy drugs from the herbal shops.

Following the preservation instructions, samples were conserved. Prior to analysis, samples were labeled by coding.

### 2.3. Sample Digestion

Samples were digested in a microwave digester using standard procedures [30, 31]. At first, approximately 3–5 g of samples was placed into individual porcelain dishes and positioned in the oven at 70°C until a constant weight was obtained. Oven-dried samples were pulverized to a fine powder. The powdered samples were preserved in plastic vials with identification marks inside the desiccator. About 1 g of homogeneous powder was taken in a Teflon vessel, and 10 mL of concentrated HNO_3_ acid (Merck, Germany) was added to in drug samples. 2 mL of 30% H_2_O_2_ (Sigma-Aldrich) was also added. The mixture was taken in a microwave digestion chamber (Milestone-Ethos One, USA), where a temperature of 180°C was applied for 15 minutes. As a result, the organic materials were decomposed from the sample matrix and metals went into solution. After digestion completion, the samples were filtered by a Whatman filter paper and quantitatively transferred to a 25 mL volumetric flask.

### 2.4. Sample Analysis

Samples were diluted and fed to an autosampler of an atomic absorption spectrophotometer (AA-7000, Shimadzu, Japan) for metal quantification, GFAAS, CVAAS, or FAAS. Cadmium, chromium, copper, iron, manganese, nickel, lead, and zinc were analyzed by flame atomic absorption spectrophotometry (FAAS); arsenic was analyzed by graphite furnace atomic absorption spectrophotometry (GFAAS); and mercury was analyzed by cold vapor atomic absorption spectrophotometry (CVAAS). The spectrophotometer was provided with single-element hollow cathode lamps and a 10-cm air-acetylene burner. Flame gas was an air-acetylene oxidizing flame. Prior to sample analysis, stock standard solutions of 1000 mg/L (Wako Chemicals, Japan) of each metal were diluted with working standards for calibration [30].

According to the requirements of the manufacturer, the spectral band-pass, wavelengths, and other instrumental conditions were set. The instrumental condition is provided in Table 1.

### 2.5. Data Validation

The data were validated by a linearity study of calibration curves and recoveries (standard recovery and spike) [32–34]. For the working standards of different metals, absorbance vs. concentration calibration curves were obtained. Coefficient of determination (*R*^2^) values were calculated for each metal from the curves. Coefficient of determination (*R*^2^) values were obtained in the range of 0.9992–0.9999 for the metals under investigation. Limits of quantifications (LOQs) of the metals were found in the range of 0.02 ppm (Hg) to 2.50 ppm (Fe). Prepared standards were rerun under the same calibration of the samples. The minimum standard recovery was 93% for manganese, and the maximum recovery was 111% for mercury. For the standard of each metal, a known amount of the respective addition was done to see the spike recovery. The minimum spike recovery was 79% for iron, and the maximum recovery was 129% for cadmium (Table 2).

### 2.6. Health Risk Assessment

Health risk assessment weighs duration and rate of intake. The process of assessing the risk to human health involves computing the relevant parameters (HQ, HI, and ILCR).

### 2.7. Noncarcinogenic Health Risk

Prolonged exposure to an individual metal can cause adverse effects on human health [35]. Adverse effects on human health were predicted by conducting risk assessments. Noncarcinogenic health risk assessment and carcinogenic health risk assessment are two such models for assessing the risks. Noncarcinogenic health risk was evaluated by determining the values of the HQ and HI [29] (Table 3). The risk assessment models rely on the computation of the risk level.

Estimated daily intake (EDI) denotes the daily consumption of toxic metals, which is computed by multiplying the concentration of metal in the sample with the ingestion rate and giving the result by body weight.

(1) $$\begin{array}{c} \text{EDI}=\frac{C\times \text{IR}}{\text{BW}}, \end{array}$$

where *C* (ppm) is the concentration of toxic metals in the antidiabetic herbal preparations, IR (kg/day) is the ingestion rate (dosage of drug taken), and BW (kg) is the body weight.

Chronic daily intake (CDI) denotes the prolonged intake of toxic metals. Under the current investigation, only oral intake is considered.

(2) $$\begin{array}{c} \text{CDI}=\frac{\text{CDI}\times \text{EF}\times \text{ED}}{\text{AT}}, \end{array}$$

where CDI (ppm/day) is the chronic daily intake. EF (days/year) is the exposure frequency, ED (years) is the exposure duration, and AT (ED × 365) (days) is the averaging time [36].

The HQ denotes the ratio of CDI to the RfD of the metal of interest.

(3) $$\begin{array}{c} \text{HQ}=\frac{\text{CDI}}{\text{RfD}}, \end{array}$$

where RfD is the reference dose that enables an individual to sustain the level of exposure over a long period of time without experiencing any harmful effects [37]. If HQ < 1, there is no concern for noncarcinogenic effects, and if HQ > 1, there is no reason for noncarcinogenic anxiety. From the literature, RfDs of different metals were found as follows: Mn (0.14 ppm/day), Fe (0.70 ppm/day), Hg (0.0001 ppm/day), Cu (0.001 ppm/day), Ni (0.02 ppm/day), Cd (0.001 ppm/day), Pb (0.004 ppm/day), Cr (0.02 ppm/day), As (0.003 ppm/day), and Zn (0.30 ppm/day) [38].

HI, an additive effect, is predicted for exposure to more than one toxic metal. With this concept, the hazard index is computed as the sum of all hazard index values for individual metals [36].

(4) $$\begin{array}{c} \text{HI}=\overset{i}{\underset{n=1}{\sum }}\text{HQ}n;i=1,2,3\ldots n, \end{array}$$

A HI value greater than 1 indicates the possibility of adverse effects on human health.

### 2.8. Carcinogenic Health Risk

Incremental Lifetime Cancer Risk (ILCR) denotes the incremental probability of a person developing any type of cancer over exposure duration as a result of daily exposure to a given daily amount of a carcinogenic element for years.

(5) $$\begin{array}{c} \text{ILCR}=\text{CDI}\times \text{CSF}, \end{array}$$

where CSF indicates the risk generated by a lifetime average of one mg/kg/day of carcinogen metal. From the literature, CSF was found to be as follows: Ni (0.84 ppm/day), Cd (6.1 ppm/day), Pb (8.5 ppm/day), and Cr (41 ppm/day). For a single carcinogenic element, the acceptable level for ILCR is 10^−6^ [39].

### 2.9. Statistical Analysis

Research data were tabulated and compiled in Microsoft Office Excel 2010. Data were further processed, calculated, and stored utilizing the same software. In the case of samples, the results of three independent replicates (*n* = 3) were represented, and the associated uncertainty was expressed by the data with a positive and negative standard deviation. Drug safety plot (histogram) was drawn by using OriginPro statistical software.

### 2.10. Map Drawing

Maps of Rajshahi City and Bangladesh were drawn using National Geographic MapMaker software. The tool provides images of the city and its suburbs, which captured a bird's-eye view. The image was cropped and edited in MS Office Word 2016 software.

## 3. Results and Discussion

### 3.1. Health Risk Assessment

#### 3.1.1. Noncarcinogenic Health Risk

HQ is used to assess the health risk of noncarcinogenic anxiety in the target population. HQ crossing unity indicates the possibility of noncarcinogenic health effects on the exposed population. HQ values ranged from 0 to 0.1630 for Mn, 0 to 4.0476 for Fe, 0 to 382.6154 for Hg, 0 to 232.1575 for Cu, 0 to 0.3534 for Ni, 0 to 0.5262 for Cd, 0 to 245.5680 for Pb, 0 to 0.2469 for Cr, 0 to 0.0965 for As, and 0 to 1.0011 for Zn (Table4). Noncarcinogenic health risk remains in 7 samples for Fe, 16 samples for Hg, 4 samples for Cu, 8 samples for Pb, and 1 sample for Zn (HQ > 1). Noncarcinogenic health risks were absent for Mn, Ni, Cd, Cr, and As in any sample under trial (HQ < 1). In agreement with this, herbal preparations exceeded unity (HQ > 1) for Cr in African investigations [40].

The effect of all metals concurrently is studied by HI values. The highest and lowest HI values were in S 17 (528.80), and S 5, S 18–S 21 (0), respectively (Table 5). 16 samples exceeded the cutoff value (HI > 1) for HI. This indicates that the health of the consumers of the formulations is at risk, and prompt intervention is required to minimize the levels of metals in the formulation to safeguard the patients from a potential health risk. In agreement with this investigation, herbal preparations exceeded the unity for HI in African investigations [40]. Contrary to our investigation, a Brazilian investigation found all HI values recorded below 1 [41].

#### 3.1.2. Carcinogenic Health Risk

As the investigated metals may give rise to the risk of both carcinogenic and noncarcinogenic anxiety, the carcinogenic risks for Pb, Cr, Cd, and Ni were also computed. Carcinogenic risk was evaluated by determining the ILCR. ILCR values for Pb, Cr, Cr, and Ni ranged from 0 to 8.35, 0 to 2.02 × 10^−01^, 0 to 3.21 × 10^−3^, and 0 to 5.94 × 10^−03^, respectively (Table 4). Among the investigated samples, 13 samples for Pb, 14 samples for Cr, 11 samples for Cd, and 14 samples for Ni pose a risk for carcinogenic anxiety (ILCR > 10^−6^).

#### 3.1.3. Comparative Study

In total, ten elemental impurities were searched in twenty-four antidiabetic herbal preparations. The metals were iron (Fe), copper (Cu), zinc (Zn), manganese (Mn), chromium (Cr), mercury (Hg), cobalt (Co), nickel (Ni), cadmium (Cd), and lead (Pb).

Mn was detected in ninety-six percent of herbal preparations (Table 6). The highest Mn was detected in S 8 (562 ± 11 ppm). The mean concentration of this metal was about five times lower (117.1 ppm) than the highest value. A nearly similar picture (Mn: 1.51–458 ppm) was obtained, while some herbal drugs were investigated by the same research group a couple of years ago [26]. The MRL for this metal is 0.26 ppm [42], which makes ninety-six percent of samples unsafe to consume. As a toxic metal, manganese toxicity affects the central nervous system (CNS) and causes damage to other organs [14]. Adverse effects on the brain and lungs were also reported upon chronic exposure to this metal [43].

Most of the antidiabetic herbal preparations were detected with Fe. While the highest Fe was detected in S 8 at a concentration as high as 59374 ± 722 ppm, the lowest Fe was detected in S 24 at a concentration as low as 24 ± 1.5 ppm (Table 6). These two values represent extreme values in iron detection, where the highest iron concentration was around 2800 times higher than the lowest detected concentration. Iron in moderate concentration was found by Ababneh [44] in the range of 6.2–1477 ppm. Acute diseases such as dizziness, nausea, vomiting, joint pain, shock, and diarrhea are related to iron overdose [45]. Another investigation conducted by the current investigators found quite the contrary in iron concentration, where samples were quantified at 10.73 ppm iron as the maximum value [46].

Hg was detected in seventy-five percent of the samples. Among them, the highest concentration was 871 ± 11 ppm in S 11 and the lowest concentration was 0.27 ± 0 ppm in S 21. Six herbal preparations were found below the detection limit of mercury (Table 6). The mean concentration of mercury was 214.9 ppm. The MRL for this metal is 0.01 ppm [42], which makes 75 percent of samples unsafe to consume. The International Conference on Harmonization (ICH) categorized the metal in class 1 in its (ICH) Q3D guideline due to the high toxicity of the metal. Involuntary, rhythmic, and oscillatory movements of a body part are observed due to the exposure of the toxic metal. Patients with cognitive impairments such as inattention, excitement, and hallucinosis were also found exposed to the metal [47]. However, instances of lower concentrations of mercury were observed by the same research group conducting the current study, where the maximum concentration never exceeded 0.05 ppm [26]. This variation of research results from region to region indicates the necessity of conducting vigorous investigation into searching for metal toxicity in herbal drugs in the country to get a clear perception of the topic being investigated. Lower instances (below 0.08–16.8 ppm) were also reported in a foreign study [48]. Ingestion of mercury in herbal supplements casts doubt on their safety. Mercury, a strong neurotoxin, can enter herbal supplements through water contamination or faulty manufacture. Long-term mercury exposure can harm the neurological system, kidneys, and heart. Regulatory agencies and producers must prioritize quality control and use extensive testing techniques to detect and eliminate mercury contamination in herbal supplements. Choose products from trusted providers that follow strict safety requirements and transparent production methods. To reduce mercury toxicity risks and maintain herbal supplement safety, this proactive strategy is necessary [49].

Cu was detected in fifty percent of the samples. The detected samples contained copper in the range of 2.38 ± 0.8–5716 ± 173 ppm, with a mean value of 253 ppm. Abruptly high concentration 5716 ± 173 ppm of S8 was nearly 50 times higher than its next lower concentration 106 ± 3 ppm of S 10 (Table 6). A similar type of investigation done by this research group with other herbal finished formulations found a similar picture of the metal concentration (4.25–49.6 ppm) if we omitted the abruptly higher highest concentration from this investigation [25]. While assessing the drugs for safety by comparing them with MRL for Cu (0.10 ppm) [42], twelve drugs were found unsafe to consume. Diseases such as arthritis, growth impairment and reproductive performance, malnutrition, irregular hair growth and depigmentation, failure of the heart, and disturbances in the gastrointestinal system are related to Cu poisoning [50]. However, an international investigation found all drugs safe to consume with a copper concentration of 0.16–0.23 ppm [9].

Ni was detected in fifty-nine percent of samples, where the metal concentration spanned from 1.46 ± 0.7 ppm (S 13) to 22.7 ± 0.9 ppm (S 5) (Table 6). The mean Ni concentrations in all studied herbal drugs were 11 ppm. The MRL for this metal is 0.60 ppm [42]. With this value, fourteen samples were found unsafe to consume. Skin allergic reactions are the most prominent adverse health effect of Ni consumption in excess. However, instances of cardiovascular disease and interference with the physiological processes of zinc and calcium were also evident [51–53]. However, in some other samples, the maximum concentration was 8.77 ppm for Ni by the research group [26]. Another investigation in Jordan by Ababnah found Ni concentrations in the range of 0.53–15.7 ppm, with a mean value of 5.44 ppm, where few samples crossed the safety limits [44].

Half of the studied samples were detected with Cd, where the highest and lowest Cd-containing samples were S 10 and S 12 with concentrations of 11.4 ± 0.5 ppm and 0.11 ± 0.5 ppm, respectively (Table 7). The average concentration of this metal was 1.4 ppm. The metal was detected in the following range in other investigations conducted elsewhere: Cd: 0.68–2.75 ppm [16], Cd: 0.48–3.08 ppm [11], Cd: 0.04–4.35 ppm [5], and Cd: 0.04–1.27 ppm [44]. All detected samples were unsafe to consume (MRL for Cd is 0.06 ppm) [42]. The ion of the metal Cd^2+^ can replace Zn^2+^, leading to cadmium toxicity. Cadmium is highly toxic and it poses toxicity at very low concentrations. The MRL of this metal is 0.06 ppm. The comparison of the detected samples with the MRL value led us to believe that all samples were unsafe for consumption. Cd can cause high blood pressure and commit the destruction of red blood cells (RBC). The metal is also known to be carcinogenic [19, 54].

Pb was detected in fifty-four percent of herbal preparations in the range of 3.91 ± 2.2 ppm (S 13)–895 ± 9 ppm (S 8) (Table 7). The mean concentration of the metal was 934.8 ppm. The MRL of this metal is 0.10 ppm [42]. With this ceiling, thirteen samples were counted as unsafe to consume in terms of metal. Another investigation left 20% of herbal drugs unsafe to consume [26]. Lead in herbal supplements can cause serious and irreversible health problems, putting public health at risk. Lead causes neurological, developmental, and reproductive disorders over time [55]. Different reproductive dysfunctions, including decreased sperm quality, disorganized epithelia, and altered sperm morphology were evident from lead poisoning [56]. Lower instances of lead, where all samples were safe to consume, were observed in two separate investigations in Turkey where data varied between 0.02 and 3.01 ppm [57] and 0.26 and 4.8 ppm [8], respectively. Polluted land, water, or inappropriate processing can cause lead contamination in herbal products. Regulators and producers must prioritize quality control, including testing, to detect and eradicate lead in herbal supplements. Consumers should choose products from trusted vendors with high quality standards. Lead-free herbal supplements are essential to the health of those who use them [55].

Around sixty percent of samples were detected with Cr, where the highest and lowest chromium-containing samples were S 10 (107 ± 2 ppm) and S 22 (2.42 ± 3.9 ppm), respectively (Table 7). Central tendency of this metal concentration is 16.6 ppm. The detected samples were unsafe to consume in terms of Cr, as the MRL was 0.05 ppm for the metal [42]. A very high concentration of chromium (maximum value of 254.15 ppm) was found in a study conducted in Poland [9]. Jurowski [6] and Tokalioglu [57] also found herbal samples contaminated with chromium with disturbing levels of chromium ranging from 4.42 to 8.74 ppm and 0.44 to 8.71 ppm, respectively, in two separate investigations conducted in European countries. A similar scenario was observed in an Arabian investigation where the chromium concentration ranged from less than 0.03 to 9.43 ppm. Cr affects different parts of the immune system, which may lead to immunostimulant or immunosuppressive effects [58]. However, some different samples (0.04 ppm) were found safe to consume in terms of chromium in another study by the same research group conducting this investigation [59].

The presence of nickel and chromium, both dangerous heavy metals, in herbal supplements gives rise to substantial concerns. Although these metals occur naturally in the environment, their buildup in herbal products could be the result of manufacturing processes or contaminated soil. The ingestion of nickel can result in detrimental physiological consequences, such as cutaneous eruptions, intestinal disturbances, and respiratory complications. A high chromium intake, on the other hand, can disrupt glucose metabolism and lead to injury to the kidneys and liver. In light of the possible health hazards linked to nickel and chromium, it is of the utmost importance that regulatory bodies and manufacturers enforce rigorous quality control protocols to safeguard herbal supplements. It is imperative that consumers maintain a state of constant vigilance and select goods from trustworthy sources, placing particular emphasis on the transparency of ingredient sourcing and the implementation of stringent testing protocols, in order to minimize the potential for encountering these perilous heavy metals [60].

As was detected in fifty-eight percent of finished formulations. The maximum concentration of the metal was found to be 6.27 ± 0.2 ppm (Table 7). All investigated samples crossed the safety endpoints (MRL for As is 0.02 ppm) [42]. This investigation is consistent with our earlier three separate investigations, where samples were collected from different sampling locations in the capital city of Bangladesh. Concentration ranges of 0.01–0.45 ppm [26], 0.01–6.74 ppm [59], and below 0.01 [46] were evident in those investigations. Arsenic poisoning in herbal supplements presents serious safety and quality problems. Herbal supplements can include arsenic from soil or faulty production. Long-term arsenic exposure can cause skin blemishes, cardiovascular difficulties, and some malignancies. To detect and prevent arsenic in herbal supplements, regulators and manufacturers must prioritize rigorous testing and quality control. Consumers should buy products from trusted sources to ensure production transparency and heavy metal testing. Public health and herbal supplement industry trust depend on this monitoring [61].

Zn was detected in ninety-two percent of herbal preparations, where the highest and lowest zinc-containing drugs were S 12 (7978 ± 112 ppm) and S 1 (0.74 ± 1.1 ppm), respectively, with a mean value of 1629.3 ppm (Table 7). The safety assessment of the drugs for the metal was done by comparing them with the MRL (15 ppm) [42]. Seventeen samples were found to be unsafe to consume. However, Zn in lower concentrations and within safe limits was observed in our recent investigation of some other herbal formulations [46]. Few samples exceeding safe limits were obtained in another investigation where the maximum concentration was 181.4 ppm [25]. In two separate international investigations, Egypt and Turkey also reported unsafe levels of Zn in herbal drugs as 15.4–73.7 ppm [15] and 3.75–88 ppm [57], respectively, for some samples. Toxic effects on blood lipoprotein levels, copper levels, and adverse effects on the immune system are evident with chronic zinc exposure exceeding the safety endpoint [62].

**Figure 2:**
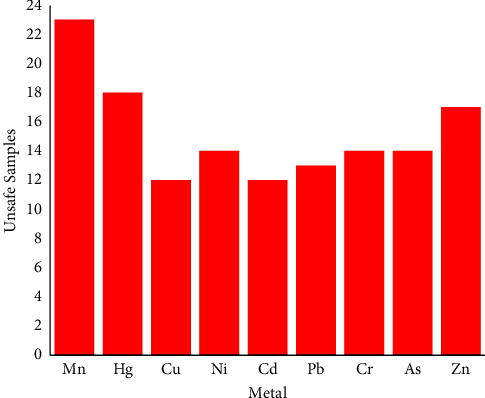
Drug safety plot for toxic metals in investigated antidiabetic herbal preparations.

To summarize the findings, a plot was constructed. In the plot, Mn was proclaimed the most concerning metal due to its exceeding levels in 23 samples (Figure 2). The concern is minimal for Cu and Cd, as their MRL crossed the safety limit in 12 probed preparations (Figure 2). Therefore, a crucial discovery for quality control of the preparations has come out in the investigation considering the prolonged exposure of metals through the formulations and their associated health risks.

#### 3.1.4. Possible Contamination Sources

**Figure 3:**
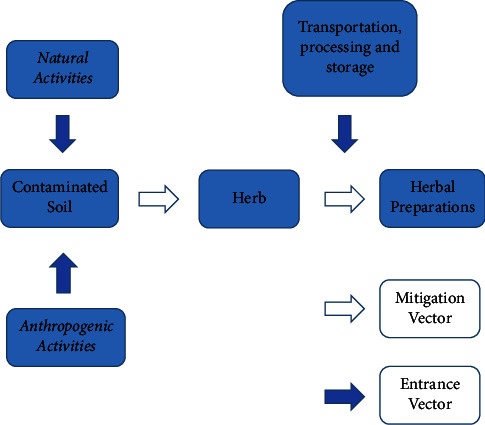
Schematic representation for entrance and mitigation of toxic metals from source to herbal preparations.

Exposure to toxic metals in herbal preparations may be due to environmental pollution and soil composition where the plants grow, which leads to metal deposition in raw herbal plants. Next, different stages of the manufacturing process also act as entry routes for metal contamination in herbal remedies if good manufacturing and laboratory practices are not well applied (Figure 3).

## 4. Conclusion

Metal contamination in drug samples is always abrupt, which poses potential human health risks. Concentrations of ten metals in different frequently sold antidiabetic herbal preparations from Rajshahi City were compared with published data and regulatory authority guidelines. Noncarcinogenic health risk remains for 50% of the metals (Fe, Hg, Cu, Pb, and Zn), where 29% of samples for Fe, 67% of samples for Hg, 17% of samples for Cu, 33% of samples for Pb, and 4% of samples for Zn crossed the acceptable levels. Noncarcinogenic health risk (HQ < 1) is absent in the remaining 50% of metals (Mn, Ni, Cd, Cr, and As). 67% of samples exceeded the acceptable value (HI > 1) for HI, implying a health risk to the consumers of the preparations. 54% of samples for Pb, 58% of samples for Cr, 46% of samples for Cd, and 58% of samples for Ni pose a risk for carcinogenic anxiety. A comparative study reveals manganese as the metal of concern the most, as its level exceeds ninety-six percent of all preparations, and Cu and Cd are the least concerning, as their MRLs did not cross the safety limit in fifty percent of drugs under investigation. This is a threat to drug safety and public health safety as well. Considering these facts, prompt interference is required to minimize the levels of metals in the formulations to protect the patients from the health risks they may face. Monitoring and measures should be taken at different points, which begins with collecting the herbs from rural areas and their vicinity to clean water sources. Pre- and postmarket monitoring should also be performed on finished herbal formulations.

## Figures and Tables

**Table 1:** Instrument operating conditions.

Metal	Sample flow rate (mL/min)	Lamp	Wavelength (nm)	Slit width (nm)	Lamp intensity (mA)
As	5	HCL	193.7	1	7
Cd	5	HCL	228.8	0.5	3
Cr	5	HCL	357.9	0.2	7
Cu	5	HCL	324.7	0.5	3
Fe	5	HCL	372	0.2	7
Pb	5	HCL	217	1	5
Mn	5	HCL	279.5	0.2	5
Co	5	HCL	240.7	0.2	7
Ni	5	HCL	232	0.2	7
Zn	5	HCL	232	0.5	5

HCL = Hollow cathode lamp.

**Table 2:** Recovery study.

Metals	Standard recovery % (*n* = 5)	Spike recovery % (*n* = 3)
As	96–103	83–106
Cd	101–104	103–129
Cr	104–110	89–119
Cu	97–109	84–111
Fe	96–104	79–91
Hg	105–111	98–115
Mn	93–105	90–97
Ni	96–100	96
Pb	98–101	93–118
Zn	95–103	118

**Table 3:** Parameters and assumptions for health risk assessment of metals through antidiabetic herbal preparations.

Parameter	Unit	Values
Metal concentration	ppm	—
Estimated daily intake (EDI)	ppm/day	—
Chronic daily intake (CDI)	ppm/day	—
Exposure frequency (EF)	Days/year	365
Exposure duration (ED)	Years	20
Body weight (BW)	Kg	65
Average time (AT)	ED × 365 days	7300

**Table 4:** Incremental lifetime cancer risk (ILCR) of metals through the consumption of antidiabetic herbal preparations.

Drug code	ILCR			
	Pb	Cr	Cd	Ni
S 1	6.95 × 10^−02^	1.43 × 10^−02^	2.57 × 10^−04^	1.02 × 10^−04^
S 2	0	3.07 × 10^−02^	0	1.53 × 10^−04^
S 3	0	8.43 × 10^−03^	1.08 × 10^−04^	1.90 × 10^−04^
S 4	4.32 × 10^−03^	0	0	0
S 5	2.21 × 10^−03^	4.60 × 10^−03^	8.48 × 10^−05^	1.42 × 10^−04^
S 6	0	5.92 × 10^−03^	0	9.75 × 10^−05^
S 7	0	0	1.98 × 10^−07^	0
S 8	1.98 × 10^−03^	0	0	7.30 × 10^−05^
S 9	0	0	0	0
S 10	0	0	0	2.98 × 10^−05^
S 11	7.09 × 10^−04^	0	0	0
S 12	1.12 × 10^−01^	3.13 × 10^−02^	0	4.73 × 10^−04^
S 13	1.30 × 10^−01^	6.73 × 10^−02^	6.23 × 10^−04^	4.93 × 10^−04^
S 14	5.21 × 10^−02^	2.10 × 10^−02^	2.11 × 10^−04^	1.22 × 10^−04^
S 15	2.31 × 10^−02^	1.04 × 10^−02^	4.27 × 10^−05^	5.08 × 10^−05^
S 16	3.09 × 10^−01^	1.68 × 10^−01^	1.90 × 10^−03^	5.94 × 10^−03^
S 17	8.35	1.61 × 10^−02^	1.40 × 10^−03^	0
S 18	0	0	0	0
S 19	0	0	0	0
S 20	0	0	0	0
S 21	0	0	0	0
S 22	1.12	2.02 × 10^−01^	3.21 × 10^−03^	7.52*E* − 04
S 23	2.81 × 10^−01^	6.93 × 10^−02^	8.91 × 10^−04^	4.89*E* − 04
S 24	0	2.51 × 10^−03^	1.36 × 10^−05^	0

**Table 5:** Hazard quotient (HQ) and hazard index (HI) of metals through the consumption of antidiabetic herbal preparations.

Drug code	HQ										HI
	Mn	Fe	Hg	Cu	Ni	Cd	Pb	Cr	As	Zn	
S 1	0.0293	0.2416	14.0985		0.0061	0.0421	2.0434	0.0174	0.0095	0.0882	16.5760
S 2	0.0212	0.0536	12.3717		0.0091			0.0374	0.0008	0.0049	12.4988
S 3	0.0239	0.0311	2.4360	0.1510	0.0113	0.0177		0.0103		0.0203	2.7016
S 4	0.0044	0.0051	0.1023				0.1269			0.0059	0.2446
S 5	0.0354	0.0220	36.8707		0.0085	0.0139	0.0651	0.0056		0.0187	37.0399
S 6	0.0312	0.0259		0.3392	0.0058			0.0072		0.0153	0.4247
S 7		0.0000				0.0000					0.0000
S 8	0.0262	0.0217	11.7868	0.1417	0.0043		0.0582		0.0046	0.0090	12.0524
S 9	0.0081	0.0429	0.8506							0.0018	0.9034
S 10	0.0031	0.0054	7.3985		0.0018					0.0024	7.4112
S 11	0.0037	0.0024	11.4586				0.0208			0.0005	11.4859
S 12	0.1042	1.1198	66.2054		0.0282		3.3059	0.0381	0.0065	0.1406	70.9486
S 13	0.0759	1.7720	10.1007	1.9591	0.0293	0.1021	3.8317	0.0821	0.0180	0.1686	18.1395
S 14	0.0343	1.4690	215.8154	0.7366	0.0073	0.0346	1.5314	0.0256	0.0062	0.0762	219.7367
S 15	0.0132	0.8078	145.6431	0.4395	0.0030	0.0070	0.6794	0.0126	0.0023	0.3365	147.9444
S 16	0.1630	3.4450	268.0615	232.16	0.3534	0.3119	9.0877	0.2051	0.0283	0.6742	514.4877
S 17	0.0238	4.0476	277.6615	0.5063		0.2292	245.568	0.0197	0.0404	0.7083	528.8049
S 18	0.0000	0.0000								0.0000	0.0000
S 19	0.0000	0.0000								0.0000	0.0000
S 20	0.0000									0.0000	0.0000
S 21	0.0000	0.0000									0.0000
S 22	0.1312	3.3421	382.6154	4.8923	0.0448	0.5262	32.8154	0.2469	0.0965	1.0011	425.7117
S 23	0.1043	3.4152	369.8400	3.9999	0.0291	0.1461	8.2694	0.0845	0.0276	0.8388	386.7548
S 24	0.0046	0.3345	90.9785	0.1878		0.0022		0.0031	0.0007	0.6077	92.1192

**Table 6:** Mn, Fe, Hg, Cu, and Ni concentrations of the investigated antidiabetic herbal preparations.

Drug code	Metal concentration (ppm)				
	Mn	Fe	Hg	Cu	Ni
S 1	12.2 ± 1.9	27.1 ± 1.4	BDL	BDL	BDL
S 2	5.06 ± 1.9	37 ± 1.3	BDL	BDL	BDL
S 3	3.82 ± 1.9	BDL	BDL	BDL	BDL
S 4	0.75 ± 1.9	33.8 ± 1.3	BDL	BDL	BDL
S 5	411 ± 9	47991 ± 291	39.08 ± 1.6	75.8 ± 3.1	22.7 ± 2
S 6	186 ± 3	39786 ± 476	835 ± 10	28.5 ± 2.3	5.64 ± 1.5
S 7	86.8 ± 1.5	26635 ± 398	686 ± 10	20.7 ± 2.5	2.85 ± 1.6
S 8	562 ± 11	59374 ± 722	660 ± 10	5716 ± 173	174 ± 2
S 9	56.5 ± 2	47960 ± 678	470 ± 10	8.57 ± 3.4	BDL
S 10	398 ± 9	50688 ± 593	829 ± 10	106 ± 3	19.4 ± 1.8
S 11	344 ± 9	56302 ± 649	871 ± 10	94.2 ± 3.1	13.7 ± 2
S 12	31.6 ± 1.9	11530 ± 13	448 ± 10	9.25 ± 3.3	BDL
S 13	61.5 ± 1.3	255 ± 3	19.8 ± 0.6	2.38 ± 0.8	1.46 ± 0.7
S 14	29.5 ± 1.3	776 ± 13	2.2 ± 0	BDL	BDL
S 15	21.8 ± 1.3	192 ± 3	37.6 ± 1.3	BDL	1.8 ± 0.7
S 16	28.3 ± 1.2	89.6 ± 1.2	62.4 ± 1.3	BDL	BDL
S 17	82.6 ± 1.3	4440 ± 30	37.5 ± 1.3	BDL	3.19 ± 0.7
S 18	115 ± 1	4738 ± 32	39.5 ± 1.4	BDL	3.41 ± 0.7
S 19	86.3 ± 1.3	1089 ± 13	35.9 ± 1.3	BDL	5.3 ± 0.7
S 20	62.5 ± 1.6	407 ± 5	4.55 ± 0	2.82 ± 2.9	4.22 ± 1.8
S 21	16.1 ± 1.6	94.8 ± 1.6	0.27 ± 0	BDL	BDL
S 22	107 ± 2	333 ± 3	79.6 ± 1.7	BDL	3.66 ± 0.9
S 23	102 ± 2	424 ± 5	BDL	7.92 ± 3.3	2.71 ± 2.1
S 24	BDL	20.9 ± 1.5	BDL	BDL	BDL
MRL	0.26	N/A	0.01	0.10	0.60

BDL = below detection limit, MRL = maximum residual limit, and N/A = not available.

**Table 7:** Cd, Pb, Cr, As, and Zn concentrations of the investigated antidiabetic herbal preparations.

Drug code	Metal concentration (ppm)				
	Cd	Pb	Cr	As	Zn
S 1	BDL	BDL	BDL	BDL	0.74 ± 1.1
S 2	BDL	BDL	BDL	BDL	1.03 ± 1.1
S 3	BDL	BDL	BDL	BDL	2.33 ± 1.1
S 4	BDL	BDL	BDL	BDL	BDL
S 5	3.95 ± 0.5	593 ± 8	63.5 ± 2.3	2.09 ± 0.1	1957 ± 27
S 6	1.34 ± 0.4	237 ± 6	19.8 ± 1.7	0.72 ± 0	885 ± 37
S 7	0.33 ± 0.4	128 ± 6	11.9 ± 11.8	0.32 ± 0	4755 ± 79
S 8	7.68 ± 0.6	895 ± 9	101 ± 3	2.09 ± 0	4980 ± 62
S 9	3.88 ± 0.5	16627 ± 169	6.66 ± 2.5	2.05 ± 0	3597 ± 55
S 10	11.4 ± 0.5	2844 ± 36	107 ± 2	6.27 ± 0.2	6507 ± 96
S 11	3.44 ± 0.5	779 ± 8	39.8 ± 2.3	1.95 ± 0.1	5926 ± 103
S 12	0.11 ± 0.5	BDL	3.01 ± 2.4	0.11 ± 0	8978 ± 112
S 13	BDL	3.91 ± 2.2	BDL	0.23 ± 0	45.2 ± 2
S 14	BDL	BDL	BDL	BDL	13.8 ± 1.1
S 15	BDL	BDL	BDL	BDL	36.9 ± 1.1
S 16	BDL	4.54 ± 2.1	BDL	BDL	7.86 ± 1
S 17	BDL	74.9 ± 2.2	4.32 ± 3	0.11 ± 0	239 ± 4
S 18	1.18 ± 0	229 ± 2	9.74 ± 3.3	0.80 ± 0	741 ± 23
S 19	BDL	BDL	21.7 ± 3.1	0.07 ± 0	42.9 ± 1.1
S 20	0.33 ± 0.5	BDL	3.84 ± 2.1	BDL	114 ± 2
S 21	BDL	13.4 ± 2.7	BDL	BDL	46.4 ± 1.3
S 22	0.30 ± 0.3	5.62 ± 2.9	2.42 ± 3.9	BDL	121 ± 3
S 23	BDL	BDL	3.37 ± 2.4	BDL	107 ± 2
S 24	0.13 ± 0.2	BDL	BDL	BDL	BDL
MRL	0.06	0.10	0.05	0.02	15

BDL = detection limit and MRL = maximum residual limit.

## Data Availability

The data used to support the findings of this study are available from the corresponding author upon request.

## References

[1] Chen L., Magliano D., Zimmet P. (2012). The worldwide epidemiology of type-2 diabetes mellitus- present and future perspectives. *Nature Reviews Endocrinology*.

[2] Bent S. (2008). Herbal medicine in the United States: review of efficacy, safety, and regulation: grand rounds at University of California, San Francisco Medical Center. *Journal of General Internal Medicine*.

[3] Padmavathi P. (2013). Drug delivery system in nano greens. *International Journal of Herbal Medicine*.

[4] Bandaranayake W. M., Ahmad I., Aqil F., Owais M. (2006). Quality control, screening, toxicity, and regulation of herbal drugs in Modern Phytomedicine. *Turning Medicinal Plants into Drugs*.

[5] Harris E. S. J., Cao S., Littlefield B. A. (2011). Heavy metal and pesticide content in commonly prescribed individual raw Chinese Herbal Medicines. *Science of the Total Environment*.

[6] Jurowski K., Krośniak M., Fołta M., Tatar B., Cole M., Piekoszewski W. (2019). Safety assessment of the trace element impurities Ni and Cr in pharmaceutical herbal products for teething from Polish pharmacies. *Biological Trace Element Research*.

[7] Alwakeel S. S. (2008). Microbial and heavy metals contamination of herbal medicines. *Research Journal of Microbiology*.

[8] Başgel S., Erdemoglu S. B. (2006). Determination of mineral and trace elements in some medicinal herbs and their infusions consumed in Turkey. *Science of the Total Environment*.

[9] Jurowski K., Fołta M., Tatar B., Berkoz M., Krośniak M. (2021). The toxicological risk assessment of Cu, Mn, and Zn as essential elemental impurities in herbal medicinal products with valerian root (valeriana officinalis L., radix) available in polish pharmacies. *Biological Trace Element Research*.

[10] Korfali S. I., Mroueh M., Al-Zein M., Salem R. (2013). Metal concentration in commonly used medicinal herbs and infusion by Lebanese population: health impact. *Journal of Food Research*.

[11] Onwordil C. T., Agbo N., Ogunwande I. A. (2015). Levels of potentially toxic metals in selected herbal medicines in Lagos, Nigeria. *Journal of Natural Sciences Research*.

[12] Saeed M., Muhammad N., Khan H. (2011). Assessment of heavy metal content of branded Pakistani herbal products. *Tropical Journal of Pharmaceutical Research*.

[13] Saper R. B., Kales S. N., Paquin J. (2004). Heavy metal content of Ayurvedic herbal medicine products. *Journal of the American Medical Association*.

[14] Sathiavelu A., Gajalakshmi S., Iswarya V., Ashwini R., Divya G., Mythili S. (2012). Evaluation of heavy metals in medicinal plants growing in. *Vellore District European Journal of Experimental Biology*.

[15] Sheded M. D., Pulford I. D., Hamed A. I. (2006). Presence of major and trace elements in seven medicinal plants growing in the South-Eastern Desert, Egypt. *Journal of Arid Environments*.

[16] Subramanian R., Gayathri S., Rathnavel C., Raj V. (2012). Analysis of mineral and heavy metals in some medicinal plants collected from local market. *Asian Pacific Journal of Tropical Biomedicine*.

[17] Who (2007). *WHO Guidelines for Assessing Quality of Herbal Medicines with Reference to Contaminants and Residues*.

[18] Springfield E. P., Eagles P. K. F., Scott G. (2005). Quality assessment of South African herbal medicines by means of HPLC finger printing. *Journal of Ethnopharmacology*.

[19] Duruibe J., Ogwuegbu M., Egwurugwu J. (2007). Heavy metal pollution and human biotoxic effects. *International Journal of the Physical Sciences*.

[20] Korfali S. I., Hawi T., Mroueh M. (2013). Evaluation of heavy metals content in dietary supplements in Lebanon. *Chemistry Central Journal*.

[21] Bethesda M. D. (1993). *US Department of Health and Human Services*.

[22] Kim H., Hughes P. J., Hawes E. M. (2014). Adverse events associated with metal contamination of traditional Chinese medicines in Korea: a clinical review. *Yonsei Medical Journal*.

[23] Mahan L., Escott-Stump S., Raymond L., Alexopoulos Y. (2012). *Krause’s Food and Nutrition Care Process*.

[24] Singh R., Gautam N., Mishra A., Gupta R. (2011). Heavy metals and living systems: an overview. *Indian Journal of Pharmacology*.

[25] Zamir R., Islam N., Faruque A. (2019). *Comparison of Toxic Metal Concentrations in Anti Diabetic Herbal Preparations (ADHPs) Available in Bangladesh Using AAS and XRF Analytical Tools, the Scientific World Journal*.

[26] Zamir R., Islam N., Rahman M. A., Rahman M. S. (2019). Daily exposure assessment of as, Ni, Hg, Al and Mn in anti diabetic herbal preparations (ADHPs). *Pharmacologyonline*.

[27] Clemett A., Amin M. M., Ara S., Akan M. M. R. (2006). Background information for Rajshahi city, Bangladesh. *WASPA Asia Project Report*.

[28] De souza I. D., Melo E. S. P., Nascimento V. A. (2021). Potential health risks of macro and micro elements in commercial medicinal plants used to treatment of diabetes. *BioMed Research International*.

[29] United States Environment Protection Agency (2021). *Risk Assessment*.

[30] Dghaim R., Al Khatib S., Rasool H., Ali Khan M. (2015). Determination of heavy metals concentration in traditional herbs commonly consumed in the United Arab Emirates. *Journal of Environmental and Public Health*.

[31] Gonzalez-Martin M. I., Revilla L., Betances-Salcedo E. V. (2018). Pesticide residues and heavy metals in commercially processed propolis. *Microchemical Journal*.

[32] Yüksel B., Kaya-Akyüzlü D., Kayaalti Z., Özdemir F., Söylemez-Gökyer D., Söylemezoglu T. (2017). Study of blood iron and vs. Blood lead levels in beta-thalassemia patients in Turkey: an application of analytical toxicology. *Atomic Spectroscopy*.

[33] Yüksel B. (2020). Quantitative GC-FID analysis of heroin for seized drugs. *The Annals of Clinical and Analytical Medicine*.

[34] Yüksel B., Öncü T., Şen N. (2023). Assessing caffeine levels in soft beverages available in Istanbul, Turkey: an LC-MS/MS application in food toxicology. *Toxicologie Analytique et Clinique*.

[35] Yüksel B., Ustaoğlu F., Yazman M. M., Şeker M. E., Öncü T. (2023). Exposure to potentially toxic elements through ingestion of canned non-alcoholic drinks sold in istanbul, türkiye: a health risk assessment study. *Journal of Food Composition and Analysis*.

[36] Zamir R., Islam N., Zahan K. E., Asraf A., Zakaria C. M. (2022). An insight into pathway and health risk assessment of toxic metals in herbal medicine. *Journal of Chemistry*.

[37] USEPA IRIS (2011). *USEPA IRIS (US Environmental Protection Agency)’s Integrated Risk Information System*.

[38] United States Environmental Protection Agency (Usepa) (2019). Regional screening level (RSL) subchronic toxicity supporting table november. https://www.epa.gov/risk/regional-screening-levels-rsls-generic-tables.

[39] Mohammadi A. A., Zarei A., Majidi S. (2019). Carcinogenic and non-carcinogenic health risk assessment of heavy metals in drinking water of Khorramabad, Iran. *MethodsX*.

[40] Meseret M., Ketema G., Kassahun H. (2020). Health risk assessment and determination of some heavy metals in commonly consumed traditional herbal preparations in northeast Ethiopia. *Journal of Chemistry*.

[41] Geronimo A. C. R., Melo E. S. P., Silva K. R. N. (2021). Human health risk assessment of heavy metals and metalloids in herbal medicines used to treat anxiety: monitoring of safety. *Frontiers in Pharmacology*.

[42] Adusei-Mensah F., Essumang D. K., Agjei R. O., Kauhanen J., Tikkanen-Kaukanen C., Ekor M. (2019). Heavy metal content and health risk assessment of commonly patronized herbal medicinal preparations from the Kumasi metropolis of Ghana. *Journal of Environmental Health Science and Engineering*.

[43] Ernst E. (2002). Toxic heavy metals and undeclared drugs in Asian herbal medicines. *Trends in Pharmacological Sciences*.

[44] Ababneh F. A. (2017). The hazard content of cadmium, lead, and other trace elements in some medicinal herbs and their water infusions. *International Journal of Analytical Chemistry*.

[45] Martin S., Griswold W. (2009). Environmental science and technology briefs for citizens. *Human health effects of heavy metals*.

[46] Zamir R., Islam N., Parveen M. (2021). Quantification and health safety assessment of some toxic metals in anti-diabetic herbal preparations collected from local retailers using the XRF analytical tool. *Biologically Active Natural Products from Asia and Africa*.

[47] Charlot J. M. (1994). Clinical Lectures of Diseases of the Nervous System. *The landmark library of neurology and neurosurgery*.

[48] Dolan S. P., Nortrup D. A., Bolger P. M., Capar S. G. (2003). Analysis of dietary supplements for arsenic, cadmium, mercury, and lead using inductively coupled plasma mass spectrometry. *Journal of Agricultural and Food Chemistry*.

[49] Yüksel D., Yuksel B., Kalafat E., Yüce T., Katlan D. C., Koç A. (2022). Assessment of lead and mercury levels in maternal blood, fetal cord blood and placenta in pregnancy with intrauterine growth restriction. *Journal of Basic and Clinical Health Sciences*.

[50] Soetan K. O., Olaiya C. O., Oyewole O. E. (2010). The importance of mineral elements for humans, domestic animals and plants: a review. *African Journal of Food Science*.

[51] Das K. K., Das S. N., Dhundasi S. A. (2008). Nickel, its adverse health effects and oxidative stress. *Indian Journal of Medical Research*.

[52] Das K. K. (2009). A comprehensive review on nickel (II) and chromium VI toxicities-possible antioxidant (allium sativum linn) defenses. *The American Journal of the Medical Sciences*.

[53] Duda-Chodak A., Baszczyk U. (2008). The impact of nickel on human health. *Journal of Elementology*.

[54] Afkhami A., Madrakian T., Siampour H. (2006). Flame atomic absorption spectrometric determination of trace quantities of cadmium in water samples after cloud point extraction in Triton X-114 without added chelating agents. *Journal of Hazardous Materials*.

[55] Yüksel B., Kayaalti Z., Kaya-Akyüzlüa D., Tekina D., Söylemezoglu T. (2016). Assessment of lead levels in maternal blood samples by graphite furnace atomic absorption spectrometry and influence of maternal blood lead on newborns. *Atomic Spectroscopy*.

[56] Ejidike I. P., Onianwa P. C. (2015). Assessment of trace metals concentration in tree barks as indicator of atmospheric pollution within Ibadan City, South-West, Nigeria. *Journal of Analytical Methods in Chemistry*.

[57] Tokalioglu S. (2012). Determination of trace elements in commonly consumed medicinal herbs by ICP-MS and multivariate analysis. *Food Chemistry*.

[58] Shrivastava R., Upreti R. K., Seth P. K., Chaturvedi U. C. (2002). Effects of chromium on the immune system. *FEMS Immunology and Medical Microbiology*.

[59] Zamir R., Islam N., Ahmed S. (2021). *Biological and Physical Contaminants in Anti- Diabetic Herbal Medicines of Bangladesh, Biologically Active Natural Products from Asia and Africa*.

[60] Yüksel B., Arıca E., Söylemezoğlu T. (2021). Assessing reference levels of nickel and chromium in cord blood, maternal blood and placenta specimens from Ankara, Turkey. *Journal of the Turkish-German Gynecological Association*.

[61] Yüksel B., Mergen G., Söylemezoğlu T. (2010). Assessment of arsenic levels in human hair by hydride generation atomic absorption spectrometry: a toxicological application. *Atomic Spectroscopy*.

[62] Fosmire G. J. (1990). Zinc toxicity. *The American Journal of Clinical Nutrition*.

